# Have COVID-19-Related Economic Shocks Affected the Health Levels of Individuals in the United States and the United Kingdom?

**DOI:** 10.3389/fpubh.2020.611325

**Published:** 2020-12-09

**Authors:** Baozhen Jiang, Zhaohui Liu, Rui Shen, Leping Huang, Yang Tong, Yuxin Xia

**Affiliations:** ^1^School of Economics, Zhejiang University of Technology, Hangzhou, China; ^2^Guangdong Key Laboratory of Stomatology, Guanghua School of Stomatology, Hospital of Stomatology, Sun Yat-sen University, Guangdong, China; ^3^School of Foreign Languages, Shenzhen University, Shenzhen, China

**Keywords:** COVID-19-related shocks, measuring health level, lockdown era, individual-level survey, unit root test with structural breaks

## Abstract

This paper introduces a health index for measuring the health level of societies during the lockdown era, i. e., for the period from March 21, 2020 to April 7, 2020. For this purpose, individual-level survey data from the Global Behaviors and Perceptions in the COVID-19 Pandemic dataset are considered. We focus on cases in the United States and the United Kingdom, and the data come from 11,270 and 11,459 respondents, respectively. We then use unit root tests with structural breaks to examine whether COVID-19-related economic shocks significantly affect the health levels of the United States and the United Kingdom. The empirical results indicate that the health levels in the United States and the United Kingdom are not significantly affected by the COVID-19-related economic shocks. The evidence shows that government directives (such as lockdowns) did not significantly change the health levels of these societies.

## Introduction

The COVID-19 pandemic invoked a coordinated response from communities, governments, institutions, and organizations to mitigate the tragedy that inexorably ensued. The onset of this global crisis was a significant shock to societies. People's attitudes toward one another may have been transformed, as exemplified by the growth in voluntary groups to support vulnerable members of the community and the Thursday-night applause for frontline healthcare workers in many countries.

The onset of the COVID-19 pandemic in early 2020 has had various policy implications related to protecting public health. Governments have imposed curfews or partial lockdowns, including closures of public spaces, schools, and workplaces, and have enacted restrictions on domestic and international travel (1, 2). However, these policy decisions have increased unemployment (3–5) and created other problems in labor markets since most workers are unable to work from home (6–8). In short, COVID-19-related economic shocks have affected societies in many different ways. Therefore, it is important to analyze whether these kinds of policy implications have significantly affected the health level of society.

In this paper, we introduce a health index for society using individual-level survey data from the Global Behaviors and Perceptions in the COVID-19 Pandemic dataset. We focus on cases in the United States and the United Kingdom, covering 11,270 and 11,459 respondents, respectively. As discussed, government policies can affect the health level of society. We also examine whether COVID-19-related shocks significantly affect the health level of the United States and the United Kingdom. At this stage, our paper focuses on the period of lockdown in the United Kingdom and some regions in the United States (March 21, 2020 to April 7, 2020). This issue allows us to determine whether government decisions (such as the lockdown) have affected the health level of these societies.

We suggest that the United Kingdom and the United States are interesting cases. Indeed, Boris Johnson and Donald Trump are seeking to restore public confidence, and they have announced various policies to mitigate the effects of the COVID-19-related economic downturn. They have announced projects, such as constructing new highways, hospitals, and schools. However, these leaders have adopted a seemingly market-oriented approach to the disease, as though discounting its existence will somehow magic it away. We also suggest that altruism may exert pressure for change to a more humanitarian approach by governments and corporate enterprises, especially in the liberal free-market economies of the United States and the United Kingdom, where economic considerations are now beginning to dominate the debate, with community health seemingly discounted. Therefore, it is noteworthy to monitor the health conditions of people living in these countries.

The remainder of this paper is organized as follows. Section Data and Methodology explains the data and the procedures of the unit root tests with structural breaks; Section 3 discusses the empirical findings; and Section 4 concludes.

## Data and Methodology

### Data

In this study, we focus on individual-level survey data covering 11,270 respondents in the United States and 11,459 respondents in the United Kingdom. The original dataset was provided by Fetzer et al. (9). The individual-level survey data of Fetzer et al. (9) are created by the snowball sampling method, which includes the survey instruments. The surveys were conducted in 68 languages, and the responses have been recorded using online tools between March 21, 2020 and April 7, 2020^1^.

At this stage, we consider the Health measure of the individual-level survey in Fetzer et al. (9). This measure asks respondents the following question, “How healthy are you?” The Health measure is defined as an index from 1 to 4, where 1 = poor, 2 = fair, 3 = good, and 4 = excellent. The scores of the responses are collapsed for each day throughout concern, and we create the health index scores in the United States and the United Kingdom, respectively. Next, we introduce the health index from 1 to 10. Naturally, a higher level on the health index means a greater health level in society.

**Figure 1:**
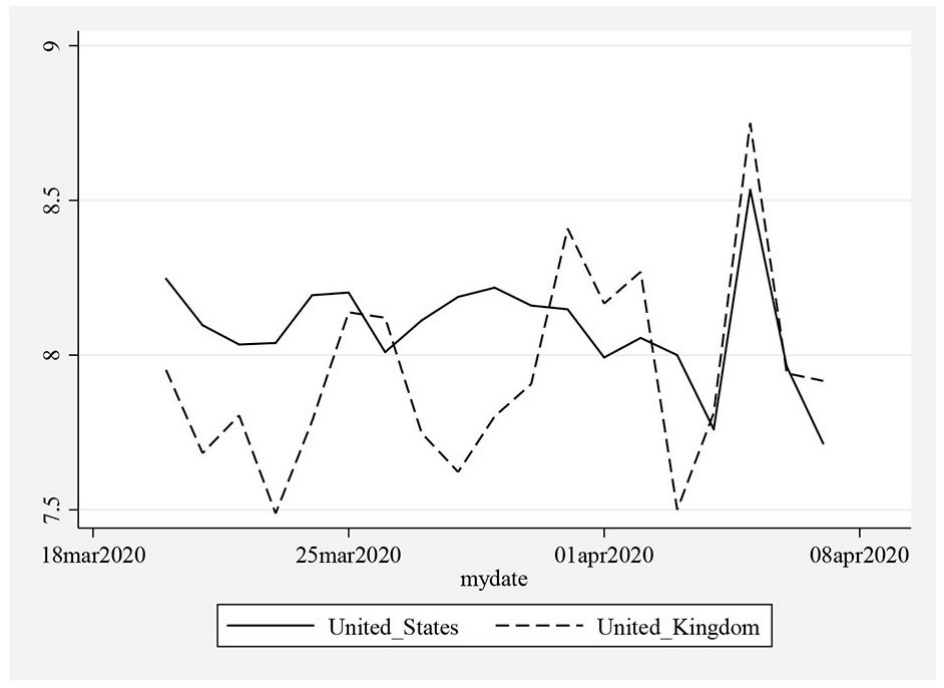
Health Levels (Index from 1 to 10) in the United Kingdom and the United States. A higher level of the index means that there is a greater health level in the society.

Figure 1 illustrates the health levels in the United States and the United Kingdom throughout concern.

In Table 1, a summary of the descriptive statistics is provided. The averages and the median values of the health index in the United States and the United Kingdom are found as 8.08 and 7.93, respectively. These results indicate that respondents in these countries live with “good health conditions.”

**Table 1:** Summary of descriptive statistics.

**Indicator**	**Definition**	**Source**	**Mean**	**Median**	**Standard deviation**	**Skewness**	**Kurtosis**	**Jarque–Bera**
Health Index_UK	Index from 0 to 10	Authors' Calculation Based on Fetzer et al. (9)	7.937	7.906	0.315	0.845	3.545	2.496 [0.2869]
Health Index_USA	Index from 0 to 10	Authors' Calculation Based on Fetzer et al. (9)	8.087	8.096	0.179	0.081	4.141	1.052 [0.5908]

*The probability values in [ ]*.

The standard deviations of the health index in the United States and the United Kingdom are observed as 0.18 and 0.31, respectively. According to the results of the ANOVA *F*-test, Satterthwaite-Welch *t*-test, *t*-test, and Welch *F*-test for equality of means between the related series, there is no statistically significant difference (at the 5% level) between the mean of health levels in the United Kingdom and the United States^2^. It is also observed that both series follow a normal distribution, and there are no issues with the non-linearity of the series.

### Unit Root Test Methodology

Following the preliminary evidence, we move on to the linear unit root tests to examine whether the external shocks have changed the pattern of the health indices in the United Kingdom and the United States. However, we should consider a unit root test with structural breaks, given the enacting of the Coronavirus Aid, Relief, and Economic Security (CARES) Act on March 27, 2020 in the United States. Therefore, we consider unit root tests that model the structural breaks in the series, which are proposed by Zivot and Andrews (10) and Perron (11).

## Empirical Findings

The results of the unit root test of Zivot and Andrews (10) are provided in Table 2.

**Table 2:** Unit root test of Zivot and Andrews (10).

**Break on the Level**	**Test Statistic and Lag**	**Break**	**Conclusion**
Health Index_UK	−4.738^***^ (4)	April 2, 2020	I(0)
Health Index_USA	−5.860^***^ (1)	April 1, 2020	I(0)
**Break on the Trend**	**Test Statistic and Lag**	**Break**	**Conclusion**
Health Index_UK	−4.924^***^ (4)	April 2, 2020	I(0)
Health Index_USA	−5.070^***^ (1)	April 1, 2020	I(0)
**Break on Both Level and Trend**	**Test Statistic and Lag**	**Break**	**Conclusion**
Health Index_UK	−4.875^***^ (4)	April 2, 2020	I(0)
Health Index_USA	−5.772^***^ (1)	April 1, 2020	I(0)

*Lags, which are selected by BIC, are in parentheses*.

*** *p < 0.01*.

*Null hypothesis: The indicator follows a unit root process*.

Table 2 reports the findings of the unit root in the level term, the time-trend term, and both the level and the time-trend terms. The optimal lags in unit root test are selected by the Bayesian Information Criteria (BIC). All results indicate that the null hypothesis, i.e., that health measures follow a unit root process, is rejected for the health measures in the United States and the United Kingdom. The Zivot–Andrews test statistics are statistically significant at the 1% level (*p* < 0.01).

We check the robustness of the findings of the unit root test of Zivot and Andrews (10). For this purpose, we also report the results of the unit root test of Perron (11) in Table 3.

**Table 3:** Unit root test of Perron (11).

**Break on the Level**	**Test Statistic and Lag**	**Break**	**Conclusion**
Health Index_UK	−5.294^***^ (2)	April 2, 2020	I(0)
Health Index_USA	−5.975^***^ (0)	April 4, 2020	I(0)
**Break on the Trend**	**Test Statistic and Lag**	**Break**	**Conclusion**
Health Index_UK	−4.851^***^ (2)	April 2, 2020	I(0)
Health Index_USA	−6.324^***^ (0)	April 1, 2020	I(0)
**Break on Both Level and Trend**	**Test Statistic and Lag**	**Break**	**Conclusion**
Health Index_UK	−6.383^***^ (2)	April 2, 2020	I(0)
Health Index_USA	−7.981^***^ (0)	April 4, 2020	I(0)

*Lags, which are selected by BIC, are in parentheses*.

*** *p < 0.01*.

*Null hypothesis: The indicator follows a unit root process*.

Table 3 provides the results of the unit root in the level term, the time-trend term, and both the level and the time-trend terms. Similarly, the optimal lags in unit root test are selected by the BIC. All findings indicate that the null hypothesis, i.e., that the health measures follow a unit root process, is rejected for the health measures in the United States and the United Kingdom. The Perron test statistics are statistically significant at the 1% level (*p* < 0.01).

Overall, the findings are robust to different unit root test techniques. Our main findings indicate that the health levels in the United States and the United Kingdom are not significantly affected by COVID-19-related economic shocks.

## Conclusion

In this study, we introduced a health index to measure the health level of society during the lockdown era, i.e., from March 21, 2020 to April 7, 2020. For this purpose, we considered individual-level survey data from the Global Behaviors and Perceptions in the COVID-19 Pandemic dataset of Fetzer et al. (9). We focused on cases in the United States and the United Kingdom, and the data come from 11,270 and 11,459 respondents, respectively. We then used the unit root tests with structural breaks of Zivot and Andrews (10) and Perron (11) to examine whether the COVID-19-related shocks significantly affected the health levels of the United States and the United Kingdom.

The empirical results show that the health levels in the United States and the United Kingdom are not significantly affected by the COVID-19-related shocks. This evidence shows that government policies (such as lockdowns) did not significantly change public health levels. However, it is important to note that there are some limitations to these results. For example, the respondents who filled out the health questionnaire could not be deceased, which means that if a family member of a deceased person filled out the questionnaire on that person's behalf, the results of the study might change. In addition, people filling in the questionnaire may be affected by government propaganda; for example, the Trump administration has been downplaying the impact of the epidemic. Respondents' assessment of their health conditions could be affected by publicity, and this would affect the results of the study.

Given that our paper focuses on the data of the early stages of the COVID-19 crisis, governments should focus their attention on the most vulnerable people in society. Arguably, increased support for the most vulnerable might be gained by more sympathetic treatment of health and other vital workers, as well as increased expenditures to improve both the quality and ease of access to medical services. Future papers can focus on the re-openings era of the COVID-19 crisis when individual-level survey data becomes available.

## Data Availability Statement

Publicly available datasets were analyzed in this study. This data can be found here: https://osf.io/3sn2k.

## Author Contributions

BJ: data curation and writing—original draft preparation. ZL: writing the original draft. RS: methodology and writing—original draft preparation. LH: investigation and software. YT: conceptualization and visualization. YX: conceptualization and supervision. All authors contributed to the article and approved the submitted version.

## Conflict of Interest

The authors declare that the research was conducted in the absence of any commercial or financial relationships that could be construed as a potential conflict of interest.

## Footnotes

^1^Refer to http://www.covid19-survey.org and Fetzer et al. (9) for detailed information.

^2^Note that there is no statistically significant difference (at the 5% level) between the median and the variances of health levels in the United Kingdom and the United States.
